# Scientific output on validation and adaptation of depression screening instruments in peruvian population

**DOI:** 10.17843/rpmesp.2022.393.11197

**Published:** 2022-09-30

**Authors:** Julio Cjuno, Andrea Moya, Eliana Calderón-Pérez, Cintia Quispe-Ilizarbe, Luis Mayon, José Livia

**Affiliations:** 1 Universidad Peruana Unión, Escuela de Posgrado, Lima, Peru. Universidad Peruana Unión Universidad Peruana Unión Escuela de Posgrado Lima Peru; 2 Universidad Peruana Unión, Escuela de Psicología, Lima, Peru. Universidad Peruana Unión Universidad Peruana Unión Escuela de Psicología Lima Peru; 3 Universidad Nacional Federico Villarreal, Facultad de Psicología, Lima, Peru. Universidad Nacional Federico Villarreal Universidad Nacional Federico Villarreal Facultad de Psicología Lima Peru

**Keywords:** Depression, Depressive Disorder, Validation, Psychometrics, Peru

## Abstract

The aim of the research was to describe the scientific output of psychometric studies on screening instruments for depression in the Peruvian population. We carried out a descriptive study of the scientific literature in Scopus, Web of Science, PubMed and SciELO, with descriptors for depression, psychometric properties, and Peru. After the review process, we included 22 studies. We found validations of screening instruments for older adults, adults, adults with depression, adult women, pregnant women, health professionals, university students, high school students and children. The Patient Health Questionnaire (PHQ-9) was the most widely used instrument. Psychometric studies cover most populations; however, native people and clinical populations remain to be studied. The PHQ-9, due to its characteristics, could be implemented in mental health policies in Peru.

## INTRODUCTION

The World Health Organization ^1^ states that by 2030 depression will be the leading cause of morbidity worldwide. The proportion of the world population with this mental health problem is estimated at 4.4%, and is more common in women than in men ^2^. In Peru, it has been reported that 15.6% of people treated in hospitals have some level of depression ^3^. It is recognized as a public health problem ^4^ ^) ^and has a significant impact on people’s quality of life ^8^. For this reason, health systems should implement or strengthen their professional services to offer psychological treatment and adapted screening instruments.

Screening and prevention tools are useful to accurately identify patients with depressive disorders and initiate treatment ^5^. There are several screening tools for depression ^6^, but they should be selected based on methodological aspects in terms of validity and reliability, considering the advances in measurement theory and its theoretical foundations ^7^. In this context, knowing the aspects of the scientific production of psychometric studies, regarding depression screening instruments in the Peruvian context, is important because it shows the process of calibration of the instruments in different age groups, geographical regions, and cultural contexts; especially in a country with a wide cultural diversity, languages, and native languages such as Peru ^8^. Therefore, this study aimed at describing the scientific output of psychometric studies on depression instruments adapted or validated in the Peruvian context.

**KEY MESSAGES**

Motivation for the study: it is necessary to identify the instruments on depression that have been adapted or validated in Peru in order to plan programs for the detection, prevention, and treatment of depression.

Main findings: 22 studies were found carried out on university students, health personnel, patients with depression, pregnant women, adults, older adults and children. No studies were found in native languages and clinical populations.

Implications: policies for the care, evaluation and follow-up of depression should consider psychometric studies for evidence-based practice.

## THE STUDY

This is a descriptive study. We searched for literature published in in PubMed, Web Of Science, Scopus and SciELO between 1993 and August 2022, either in Spanish or English. The search strategy included terms and descriptors for depression ^9^, psychometric properties and Peruvian population; the system was validated by an expert in bibliometrics for better search precision (Supplementary Material).

The search was conducted by four independent reviewers, the results were organized in Microsoft Excel 2016. We found 200 results in Scopus, 158 in Web Of Science, 132 in PubMed and 47 in SciELO. The titles and abstracts were reviewed, seeking to include studies that aimed to validate or adapt depression assessment instruments in Peruvian populations, either as a primary or secondary objective. Original articles, brief reports, short communications, and letters to the editor were selected. Fifteen studies were eligible from Scopus, 10 from Web Of Science, 8 from SciELO (core collection) and 3 from PubMed. After eliminating duplicate items, 22 studies were selected. Works considered as gray literature (undergraduate and graduate theses and studies published in journals with indexing different from the databases mentioned) were not included (Supplementary material).

Once the list of selected articles was obtained, we carried out the analysis of the complete document and compiled the data. The variables considered were: year of publication, type of article, language, quartile of the journal according to Scimago Journal & Country Rank at the date of data collection, country of the corresponding author, affiliation of the corresponding author, city of the Peruvian corresponding author, profession of the Peruvian corresponding author at the time of data collection according to the registry of degrees and titles of the National Superintendence of University Higher Education (SUNEDU), funding of the study, scientific output for ten-year periods, population in which the instrument was evaluated, language of origin and target language in the translations of the instruments and psychometric properties evaluated.

The statistical analysis was descriptive; frequencies and percentages were estimated because the variables were categorical. We used the SPSS version 26 statistical program. 

Regarding ethical aspects, the principle of scientific integrity was respected, since the study analyzed scientific articles published in peer-reviewed journals.

## FINDINGS

When assessing the scientific output on validation or adaptation of instruments that assess depression in the Peruvian population, we found that 12 articles (54.6%) were published in Spanish; 20 (91.0%) were original articles; 9 (40.9%) were published in Q1 journals and 10 (45.0%) in English-speaking journals. Likewise, 15 (68.2%) studies were led by Peruvian researchers, of which 5 (22.8%) were affiliated with the Universidad Peruana Cayetano Heredia; 13 (59.1%) Peruvian correspondent researchers reside in Lima, in which physicians specializing in psychiatry (5) and psychologists ^5^ took the lead, each accounting for 22.7%. In addition, 15 (68.2%) studies came from research projects funded by the researchers; 16 (72.7%) were published between 2013 and 2022, and 17 (77.2%) studies were published in international journals (Table 1).

**Table 1:** Characteristics of psychometric scientific production on depression in Peru (n=22).

Variables	n	%
Document language		
Spanish	12	54.6
English	9	40.9
English and Spanish	1	4.5
Type of document		
Original article	20	91.0
Brief report	1	4.5
Letter to the editor	1	4.5
Quartile of the journal ^a^		
Q1	9	40.9
Q3	3	13.6
Q2	2	9.1
Q4	2	9.1
No quartile	6	27.3
Country of the corresponding author		
Peru	14	63.7
United States	6	27.3
Colombia	1	4.5
Norway	1	4.5
Affiliation of the corresponding author		
Universidad Peruana Cayetano Heredia	5	22.8
Harvard School of Public Health	4	18.3
Universidad de San Martín de Porres	3	13.7
Universidad de Boston	2	9.1
Universidad Privada del Norte	2	9.1
Instituto Nacional de Salud del Perú	1	4.5
Temple University	1	4.5
Universidad Católica Santo Toribio de Mogrovejo	1	4.5
Universidad Científica del Sur	1	4.5
Universidad Nacional Mayor de San Marcos	1	4.5
Universidad Norbert Wiener	1	4.5
City of the Peruvian corresponding author		
Lima	13	59.1
Chiclayo	1	4.5
Cajamarca	1	4.5
Corresponding author with another nationality	7	31.9
Profession of the Peruvian corresponding author		
Physician specialized in psychiatry	5	22.7
Psychologist	5	22.7
Physician with other specialty	3	13.6
Physician with a master’s degree in epidemiology	2	9.1
Corresponding author with another nationality	7	31.9
National or international funding		
No	15	68.2
Yes	7	31.8
Scientific output for 10-year periods		
2013 to 2022	16	72.7
2003 to 2012	5	22.8
1993 to 2002	1	4.5

a According to the Scimago Journal & Country Rank 2022

Validations or adaptations were carried out on adults (4 of 22); adults with depression (3 of 22); adult women (4 of 22); pregnant women (4 of 22); health professionals (1 of 22); college students (2 of 22); high school students (1 of 22); and children (1 of 22). In addition, 17 (72.2%) of the validations or adaptations were conducted on nonclinical samples, and most instruments (13 of 22) were translated from English to Spanish (Table 2).

**Table 2:** Population where the adaptation was carried out and source and target language of translation.

Variables	n	%
Population in which the instrument was evaluated		
Adults	4	18.3
Adult women	4	18.3
Pregnant women	4	18.3
Adults with depression	3	13.6
University students	2	9.0
Older adults	1	4.5
High school students	1	4.5
Children	1	4.5
Healthcare professionals	1	4.5
Youth, adults, seniors	1	4.5
Source and target language (translation)		
English - Spanish (Peru)	13	59.1
Constructed in Spanish (Peru)	6	27.3
Spanish (Spain) - Spanish (Peru)	2	9.1
Spanish (Colombia) - Spanish (Peru)	1	4.5

The psychometric studies analyzed reliability (20 of 22); construct validity (20 of 22); sensitivity and specificity (15 of 22) (Table 3). On the other hand, most studies (9 out of 22) adapted the Patient Health Questionnaire (PHQ-9) in university students, health personnel, pregnant women, adults, and older adults (Table 4).

**Table 3:** Psychometric properties evaluated in the instruments that measure depression in Peru.

Psychometric properties	Yes		No	
	n	%	n	%
Construct validity	20	90.9	2	9.1
Reliability	20	90.9	2	9.1
Sensitivity and specificity	15	68.2	7	31.8
Content validity	9	40.9	13	59.1
Invariance analysis	4	18.3	18	81.7

**Table 4:** Instruments adapted to assess depression in Peru.

Instruments ^a^	n
Patient Health Questionnaire (PHQ-9)	9
Edinburgh Postnatal Depression Scale (EPDS)	2
Inventario de depresión de Beck (BDI-II)	2
State-Trait Depression Inventory (ST-DEP)	2
Patient Health Questionnaire (PHQ-2)	1
Escala de depresión Geriátrica GDS-4	1
General Health Questionnaire (GHQ-12)	1
Children’s Depression Inventory-short (CDI)	1
The Self-Reporting Questionnaire (SRQ)	1
Depressive Psychopathology Scale (DPS)	1
Center for Epidemiological Studies Depression Scale (CES-D)	1
Zung Self-Rating Depression Scale (ZSDS)	1

a There are 23 screening instruments in the Peruvian context because some studies validated two instruments and compared them.

## DISCUSSION

In the last ten years (2013 - 2022), most studies adapting or validating screening instruments for depression were found in English-language, quartile 1 journals. The preference for international, English-language, quartile 1 journals may be due to researchers seeking the highest visibility and impact of their work in the scientific community ^10^.

International scientific journals may be preferred by researchers because Peruvian scientific journals have not yet reached the first or second quartile ^11^. However, there has been an increased interest in the adaptation and validation of depression screening tests, possibly driven by the quality policies of Peruvian universities that started hiring researchers as part of the teaching staff ^12^ and licensing process. 

Instruments have been validated in populations of older adults, adults, adults with depression, adult women, pregnant women, health professionals, university students, high school students and children, reflecting the diversity of the age groups. A pending task is to adapt or construct instruments in native languages, because our country, due to its cultural and linguistic richness, has more than 50 native languages ^13^, a cultural and linguistic difference that starts from the same theoretical construction of how this population perceives depression; therefore, instruments adapted to this context are needed ^7^. Another reason to encourage the adaptation of instruments to native people is the prevalence of depression in Quechua people, which reaches 38.9% ^9^, and is seven times higher than the worldwide prevalence in adults ^1^.

Most studies showed reliability, construct validity, sensitivity/specificity, and content validity analyses. The validity and reliability of the studies show that the appropriate statistical and psychometric processes are being followed for the adaptation and construction of instruments ^7^, since content validity makes it possible to adapt or correct the wording to the cultural-linguistic context and evaluates the relevance of the content related to the theoretical construct ^14^. Construct validity analyzes the factors that explain the construct from its internal structure, reliability determines how accurate the scores of the instrument are under certain conditions, while specificity and sensitivity determine the diagnostic capacity with which an instrument will efficiently score among participants who have or do not have the problem being assessed ^14^.

Instruments that do not imply biases are necessary due to bio-sociodemographic diversity of the Peruvian population; therefore, the analysis of invariance is important, even if only a few instruments have this characteristic. This procedure verifies whether the instrument could be transversal to the characteristics of the population under study, such as age groups, sex, place of residence, clinical/non-clinical populations, and other similar ones ^7^. Therefore, not all instruments would be suitable for use in different populations or groups.

Most adaptation or validation studies analyzed the PHQ-9, which can be used in university students, health personnel, adults of both sexes, pregnant women in primary health care, and older adults. This preference may be due to its adequate psychometric properties and adaptations to more than 20 languages, being the most widely used screening instrument worldwide and chosen in an international consensus among 15 instruments evaluated for depression screening ^15^. In Peru, the PHQ-9 has been culturally adapted by experts, and its validity, reliability and invariance have been analyzed ^16^, which determines its eligibility over other instruments.

In this context, Peruvian agencies responsible for mental health care could implement the PHQ-9 in their depression care guidelines in populations where it already has solid psychometric evidence. Especially, because some official documents such as the technical guideline for mental health care of health personnel in the context of COVID-19 of the Ministry of Health, recommends the Self-Reporting Questionnaire (SRQ) ^17^ which, to date, has been validated only in women, while the Hamilton Depression Rating Scale (HDRS) ^18^ is recommended for screening in primary health care, with no evidence of validation in Peru, and the Abbreviated Yesavage Scale ^19^ is implemented in the Technical Health Standard for the Comprehensive Health Care of the Elderly ^19^. The latter is not recommended for use because even though it has initial validity of its internal structure, it lacks reliability ^20^. This is of concern, since the selection of screening instruments should be made considering the best evidence of their psychometric properties.

A limitation of this study is that we did not include publications from journals indexed in other regional databases or from the gray literature. However, by searching Scopus, Web of Science, PubMed and SciELO, we included the largest refereed literature on the validity or construction of tests assessing depression in the Peruvian population.

In conclusion, we found 22 studies including adaptations and validations of instruments that assess depression in the Peruvian population. They have been adapted for university students, health personnel, patients with depression, pregnant women, adults of both sexes, older adults, and children. Instrumental studies in native languages and in clinical populations are still needed. Due to its characteristics, the PHQ-9 could be implemented in policies of care, evaluation, and follow-up of depression in populations where psychometric studies have been carried out.
