# Supplementary material for: Scientific output on validation and adaptation of depression screening instruments in peruvian population
Source: Rev Peru Med Exp Salud Publica. 2022 Sep 30;39(3):357–61. doi: 10.17843/rpmesp.2022.393.11197 (PMC11397671; doi:10.17843/rpmesp.2022.393.11197)

**MATERIAL SUPLEMENTARIO**

**Anexo 1.**

**ESTRATEGIA DE BÚSQUEDA PARA PUBMED**

(Depression[mesh] OR depression[tiab] OR Depressive*[tiab] OR "Depressive Disorder*"[tiab] OR "Depressive symptom*"[tiab] OR “postpartum depression”[tiab] OR “Treatment-resistant depression”[tiab] OR “major depression”[tiab] OR "Bipolar Disorder"[tiab] OR "Adjustment Disorders"[tiab] OR "Psychiatric Status Rating Scales”[tiab] OR "Psychotic Affective Disorders"[tiab] OR "Perry syndrome"[tiab] OR “Major depressive disorder”[tiab] OR disthymi*[tiab] OR "mood disorder"[tiab] OR dysphoria[tiab] OR melancholia[tiab] OR "mourning syndrome"[tiab] OR pseudodementia[tiab] OR "mourning syndrome"[tiab] OR "seasonal affective disorder"[tiab] OR "depressive psychosis"[tiab] OR "premenstrual dysphoric disorder"[tiab] OR "mood disorder"[tiab] OR "Depression Anxiety"[tiab] OR "puerperal depression"[tiab] OR "Adjustment Disorders"[tiab] OR "maternal depression"[tiab] OR "late life depression"[tiab] OR "Depression Scale"[tiab] OR "antepartum depression"[tiab] OR "Depression, Postpartum"[tiab] OR "Depressive Disorder, Treatment-Resistant"[tiab] OR "Depressive Disorder, Major"[tiab] OR "Affective Disorders, Psychotic"[tiab] OR "Major Depressive Disorder 1"[tiab] OR "Major Depressive Disorder 2"[tiab]) AND (“psychometrics”[MeSH] OR psychometr*[tiab] OR “Health Status Indicators”[Mesh] OR “reproducibility of results”[MeSH] OR reproducib*[tiab] OR “discriminant analysis”[MeSH] OR reliab*[tiab] OR unreliab*[tiab] OR valid*[tiab] OR coefficient[tiab] OR homogene*[tiab] OR “internal consistency”[tiab] OR (cronbach*[tiab] AND (alpha[tiab] OR alphas[tiab])) OR (item[tiab] AND (correlation*[tiab] OR selection*[tiab] OR reduction*[tiab])) OR “precise values”[tw] OR (reliab*[tiab] AND (test[tiab] OR retest[tiab])) OR stability[tiab] OR interrater[tiab] OR inter-rater[tiab] OR intrarater[tiab] OR intra-rater[tiab] OR kappa[tiab] OR generaliza*[tiab] OR generalisa*[tiab] OR concordance[tiab] OR (intraclass[tiab] AND correlation*[tiab]) OR discriminative[tiab] OR “known group”[tiab] OR “factor analys*”[tiab] OR “factor structure*”[tiab] OR dimension*[tiab] OR subscale*[tiab] OR error*[tiab] OR (variability[tiab] AND (analysis[tiab] OR values[tiab])) OR “standard error of measurement”[tiab] OR sensitiv*[tiab] OR responsive*[tiab] OR interpretab*[tiab] OR ((minimal[tiab] OR minimally[tiab]) AND (significant[tiab] OR detectable[tiab]) AND (difference[tiab])) OR “ceiling effect”[tiab] OR “floor effect”[tiab] OR IRT[tiab] OR Rasch[tiab] OR “Differential item functioning”[tiab] OR DIF[tiab] OR “item bank”[tiab] OR “cross-cultural equivalence”[tiab] OR Adaptation[tiab] OR "Cross-Cultural Adaptation"[tiab] OR translati*[tiab] OR translation-back[tiab] OR back-translation[tiab] OR "back translation"[tiab] OR "forward translation"[tiab] OR forward-translation[tiab] OR "backward translation"[tiab] OR propert*[tiab] OR invariance*[tiab] OR "positive predictive value"[tiab] OR "negative predictive value"[tiab]) AND (Peruvian[tiab] OR Peruvians[tiab] OR Peru[tiab])

**ESTRATEGIA DE BÚSQUEDA PARA WEB OF SCIENCE**

(TI=(depression OR depressive* OR "Depressive Disorder*" OR "Depressive symptom*" OR "postpartum depression" OR "Treatment-resistant depression" OR "major depression" OR "Bipolar Disorder" OR "Adjustment Disorders" OR "Psychiatric Status Rating Scales" OR "Psychotic Affective Disorders" OR "Perry syndrome" OR "Major depressive disorder" OR disthymi* OR "mood disorder" OR dysphoria OR melancholia OR "mourning syndrome" OR pseudodementia OR "mourning syndrome" OR "seasonal affective disorder" OR "depressive psychosis" OR "premenstrual dysphoric disorder" OR "mood disorder" OR "Depression Anxiety" OR "puerperal depression" OR "Adjustment Disorders" OR "maternal depression" OR "late life depression" OR "Depression Scale" OR "antepartum depression" OR "Depression, Postpartum" OR "Depressive Disorder, Treatment-Resistant" OR "Depressive Disorder, Major" OR "Affective Disorders, Psychotic" OR "Major Depressive Disorder 1" OR "Major Depressive Disorder 2") OR AB=(depression OR depressive* OR "Depressive Disorder*" OR "Depressive symptom*" OR "postpartum depression" OR "Treatment-resistant depression" OR "major depression" OR "Bipolar Disorder" OR "Adjustment Disorders" OR "Psychiatric Status Rating Scales" OR "Psychotic Affective Disorders" OR "Perry syndrome" OR "Major depressive disorder" OR disthymi* OR "mood disorder" OR dysphoria OR melancholia OR "mourning syndrome" OR pseudodementia OR "mourning syndrome" OR "seasonal affective disorder" OR "depressive psychosis" OR "premenstrual dysphoric disorder" OR "mood disorder" OR "Depression Anxiety" OR "puerperal depression" OR "Adjustment Disorders" OR "maternal depression" OR "late life depression" OR "Depression Scale" OR "antepartum depression" OR "Depression, Postpartum" OR "Depressive Disorder, Treatment-Resistant" OR "Depressive Disorder, Major" OR "Affective Disorders, Psychotic" OR "Major Depressive Disorder 1" OR "Major Depressive Disorder 2") OR AK=(depression OR depressive* OR "Depressive Disorder*" OR "Depressive symptom*" OR "postpartum depression" OR "Treatment-resistant depression" OR "major depression" OR "Bipolar Disorder" OR "Adjustment Disorders" OR "Psychiatric Status Rating Scales" OR "Psychotic Affective Disorders" OR "Perry syndrome" OR "Major depressive disorder" OR disthymi* OR "mood disorder" OR dysphoria OR melancholia OR "mourning syndrome" OR pseudodementia OR "mourning syndrome" OR "seasonal affective disorder" OR "depressive psychosis" OR "premenstrual dysphoric disorder" OR "mood disorder" OR "Depression Anxiety" OR "puerperal depression" OR "Adjustment Disorders" OR "maternal depression" OR "late life depression" OR "Depression Scale" OR "antepartum depression" OR "Depression, Postpartum" OR "Depressive Disorder, Treatment-Resistant" OR "Depressive Disorder, Major" OR "Affective Disorders, Psychotic" OR "Major Depressive Disorder 1" OR "Major Depressive Disorder 2")) AND (TI=(psychometr* OR "Health Status Indicators" OR reproducib* OR "discriminant analysis" OR reliab* OR unreliab* OR valid* OR coefficient OR homogene* OR "internal consistency" OR ( cronbach* AND ( alpha* ) ) OR ( item AND ( correlation* OR selection* OR reduction* ) ) OR "precise values" OR ( reliab* AND ( test OR retest ) ) OR stability OR interrater OR inter-rater OR intrarater OR intra-rater OR kappa* OR generaliza* OR generalisa* OR concordance OR ( intraclass AND correlation* ) OR discriminative OR "known group" OR "factor analys*" OR "factor structure*" OR dimension* OR subscale* OR error* OR ( variability AND ( analysis OR values ) ) OR "standard error of measurement" OR sensitiv* OR responsive* OR interpretab* OR ( ( minimal*) AND (significant OR detectable ) AND (difference ) ) OR ( small* AND ( real OR detectable )) OR "ceiling effect" OR "floor effect" OR irt OR rasch OR "Differential item functioning" OR dif OR "item bank" OR "cross-cultural equivalence" OR adaptation OR "Cross-Cultural Adaptation" OR translati* OR translation-back OR back-translation OR "back translation" OR "forward translation" OR forward-translation OR "backward translation" OR propert* OR invariance* OR "positive predictive value" OR "negative predictive value”) OR AB=(psychometr* OR "Health Status Indicators" OR reproducib* OR "discriminant analysis" OR reliab* OR unreliab* OR valid* OR coefficient OR homogene* OR "internal consistency" OR ( cronbach* AND ( alpha* ) ) OR ( item AND ( correlation* OR selection* OR reduction* ) ) OR "precise values" OR ( reliab* AND ( test OR retest ) ) OR stability OR interrater OR inter-rater OR intrarater OR intra-rater OR kappa* OR generaliza* OR generalisa* OR concordance OR ( intraclass AND correlation* ) OR discriminative OR "known group" OR "factor analys*" OR "factor structure*" OR dimension* OR subscale* OR error* OR ( variability AND ( analysis OR values ) ) OR "standard error of measurement" OR sensitiv* OR responsive* OR interpretab* OR ( ( minimal*) AND (significant OR detectable ) AND (difference ) ) OR ( small* AND ( real OR detectable )) OR "ceiling effect" OR "floor effect" OR irt OR rasch OR "Differential item functioning" OR dif OR "item bank" OR "cross-cultural equivalence" OR adaptation OR "Cross-Cultural Adaptation" OR translati* OR translation-back OR back-translation OR "back translation" OR "forward translation" OR forward-translation OR "backward translation" OR propert* OR invariance* OR "positive predictive value" OR "negative predictive value”) OR AK=(psychometr* OR "Health Status Indicators" OR reproducib* OR "discriminant analysis" OR reliab* OR unreliab* OR valid* OR coefficient OR homogene* OR "internal consistency" OR ( cronbach* AND ( alpha* ) ) OR ( item AND ( correlation* OR selection* OR reduction* ) ) OR "precise values" OR ( reliab* AND ( test OR retest ) ) OR stability OR interrater OR inter-rater OR intrarater OR intra-rater OR kappa* OR generaliza* OR generalisa* OR concordance OR ( intraclass AND correlation* ) OR discriminative OR "known group" OR "factor analys*" OR "factor structure*" OR dimension* OR subscale* OR error* OR ( variability AND ( analysis OR values ) ) OR "standard error of measurement" OR sensitiv* OR responsive* OR interpretab* OR ( ( minimal*) AND (significant OR detectable ) AND (difference ) ) OR ( small* AND ( real OR detectable )) OR "ceiling effect" OR "floor effect" OR irt OR rasch OR "Differential item functioning" OR dif OR "item bank" OR "cross-cultural equivalence" OR adaptation OR "Cross-Cultural Adaptation" OR translati* OR translation-back OR back-translation OR "back translation" OR "forward translation" OR forward-translation OR "backward translation" OR propert* OR invariance* OR "positive predictive value" OR "negative predictive value”)) AND (TI=(Peruvian OR Peruvians OR Peru ) OR AB=(Peruvian OR Peruvians OR Peru ) OR AK=(Peruvian OR Peruvians OR Peru ))

**ESTRATEGÍA DE BÚSQUEDA PARA SCOPUS**

( TITLE ( depression OR depressive* OR "Depressive Disorder*" OR "Depressive symptom*" OR "postpartum depression" OR "Treatment-resistant depression" OR "major depression" OR "Bipolar Disorder" OR "Adjustment Disorders" OR "Psychiatric Status Rating Scales" OR "Psychotic Affective Disorders" OR "Perry syndrome" OR "Major depressive disorder" OR disthymi* OR "mood disorder" OR dysphoria OR melancholia OR "mourning syndrome" OR pseudodementia OR "mourning syndrome" OR "seasonal affective disorder" OR "depressive psychosis" OR "premenstrual dysphoric disorder" OR "mood disorder" OR "Depression Anxiety" OR "puerperal depression" OR "Adjustment Disorders" OR "maternal depression" OR "late life depression" OR "Depression Scale" OR "antepartum depression" OR "Depression, Postpartum" OR "Depressive Disorder, Treatment-Resistant" OR "Depressive Disorder, Major" OR "Affective Disorders, Psychotic" OR "Major Depressive Disorder 1" OR "Major Depressive Disorder 2" ) OR ABS ( depression OR depressive* OR "Depressive Disorder*" OR "Depressive symptom*" OR "postpartum depression" OR "Treatment-resistant depression" OR "major depression" OR "Bipolar Disorder" OR "Adjustment Disorders" OR "Psychiatric Status Rating Scales" OR "Psychotic Affective Disorders" OR "Perry syndrome" OR "Major depressive disorder" OR disthymi* OR "mood disorder" OR dysphoria OR melancholia OR "mourning syndrome" OR pseudodementia OR "mourning syndrome" OR "seasonal affective disorder" OR "depressive psychosis" OR "premenstrual dysphoric disorder" OR "mood disorder" OR "Depression Anxiety" OR "puerperal depression" OR "Adjustment Disorders" OR "maternal depression" OR "late life depression" OR "Depression Scale" OR "antepartum depression" OR "Depression, Postpartum" OR "Depressive Disorder, Treatment-Resistant" OR "Depressive Disorder, Major" OR "Affective Disorders, Psychotic" OR "Major Depressive Disorder 1" OR "Major Depressive Disorder 2" ) OR KEY ( depression OR depressive* OR "Depressive Disorder*" OR "Depressive symptom*" OR "postpartum depression" OR "Treatment-resistant depression" OR "major depression" OR "Bipolar Disorder" OR "Adjustment Disorders" OR "Psychiatric Status Rating Scales" OR "Psychotic Affective Disorders" OR "Perry syndrome" OR "Major depressive disorder" OR disthymi* OR "mood disorder" OR dysphoria OR melancholia OR "mourning syndrome" OR pseudodementia OR "mourning syndrome" OR "seasonal affective disorder" OR "depressive psychosis" OR "premenstrual dysphoric disorder" OR "mood disorder" OR "Depression Anxiety" OR "puerperal depression" OR "Adjustment Disorders" OR "maternal depression" OR "late life depression" OR "Depression Scale" OR "antepartum depression" OR "Depression, Postpartum" OR "Depressive Disorder, Treatment-Resistant" OR "Depressive Disorder, Major" OR "Affective Disorders, Psychotic" OR "Major Depressive Disorder 1" OR "Major Depressive Disorder 2" ) ) AND ( TITLE ( psychometr* OR "Health Status Indicators" OR reproducib* OR "discriminant analysis" OR reliab* OR unreliab* OR valid* OR coefficient OR homogene* OR "internal consistency" OR ( cronbach* AND ( alpha* ) ) OR ( item AND ( correlation* OR selection* OR reduction* ) ) OR "precise values" OR ( reliab* AND ( test OR retest ) ) OR stability OR interrater OR inter-rater OR intrarater OR intra-rater OR kappa* OR generaliza* OR generalisa* OR concordance OR ( intraclass AND correlation* ) OR discriminative OR "known group" OR "factor analys*" OR "factor structure*" OR dimension* OR subscale* OR error* OR ( variability AND ( analysis OR values ) ) OR "standard error of measurement" OR sensitiv* OR responsive* OR interpretab* OR ( ( minimal* ) AND ( significant OR detectable ) AND ( difference ) ) OR ( small* AND ( real OR detectable ) ) OR "ceiling effect" OR "floor effect" OR irt OR rasch OR "Differential item functioning" OR dif OR "item bank" OR "cross-cultural equivalence" OR adaptation OR "Cross-Cultural Adaptation" OR translati* OR translation-back OR back-translation OR "back translation" OR "forward translation" OR forward-translation OR "backward translation" OR propert* OR invariance* OR "positive predictive value" OR "negative predictive value" ) OR ABS ( psychometr* OR "Health Status Indicators" OR reproducib* OR "discriminant analysis" OR reliab* OR unreliab* OR valid* OR coefficient OR homogene* OR "internal consistency" OR ( cronbach* AND ( alpha* ) ) OR ( item AND ( correlation* OR selection* OR reduction* ) ) OR "precise values" OR ( reliab* AND ( test OR retest ) ) OR stability OR interrater OR inter-rater OR intrarater OR intra-rater OR kappa* OR generaliza* OR generalisa* OR concordance OR ( intraclass AND correlation* ) OR discriminative OR "known group" OR "factor analys*" OR "factor structure*" OR dimension* OR subscale* OR error* OR ( variability AND ( analysis OR values ) ) OR "standard error of measurement" OR sensitiv* OR responsive* OR interpretab* OR ( ( minimal* ) AND ( significant OR detectable ) AND ( difference ) ) OR ( small* AND ( real OR detectable ) ) OR "ceiling effect" OR "floor effect" OR irt OR rasch OR "Differential item functioning" OR dif OR "item bank" OR "cross-cultural equivalence" OR adaptation OR "Cross-Cultural Adaptation" OR translati* OR translation-back OR back-translation OR "back translation" OR "forward translation" OR forward-translation OR "backward translation" OR propert* OR invariance* OR "positive predictive value" OR "negative predictive value" ) OR KEY ( psychometr* OR "Health Status Indicators" OR reproducib* OR "discriminant analysis" OR reliab* OR unreliab* OR valid* OR coefficient OR homogene* OR "internal consistency" OR ( cronbach* AND ( alpha* ) ) OR ( item AND ( correlation* OR selection* OR reduction* ) ) OR "precise values" OR ( reliab* AND ( test OR retest ) ) OR stability OR interrater OR inter-rater OR intrarater OR intra-rater OR kappa* OR generaliza* OR generalisa* OR concordance OR ( intraclass AND correlation* ) OR discriminative OR "known group" OR "factor analys*" OR "factor structure*" OR dimension* OR subscale* OR error* OR ( variability AND ( analysis OR values ) ) OR "standard error of measurement" OR sensitiv* OR responsive* OR interpretab* OR ( ( minimal* ) AND ( significant OR detectable ) AND ( difference ) ) OR ( small* AND ( real OR detectable ) ) OR "ceiling effect" OR "floor effect" OR irt OR rasch OR "Differential item functioning" OR dif OR "item bank" OR "cross-cultural equivalence" OR adaptation OR "Cross-Cultural Adaptation" OR translati* OR translation-back OR back-translation OR "back translation" OR "forward translation" OR forward-translation OR "backward translation" OR propert* OR invariance* OR "positive predictive value" OR "negative predictive value" ) ) AND ( TITLE ( peruvian OR peruvians OR peru ) OR ABS ( peruvian OR peruvians OR peru ) OR KEY ( peruvian OR peruvians OR peru ) )

**ESTRATEGIA DE BÚSQUEDA PARA SCIELO**

psychometric OR "Health Status Indicators" OR reproducibility OR reliability OR "discriminant analysis" OR coefficient OR homogeneity OR "internal consistency" OR cronbach OR alpha OR "precise values" OR stability OR interrater OR inter-rater OR intrarater OR intra-rater OR kappa OR generalizability OR discriminative OR "known group" OR "factor analys" OR "factor structure" OR "standard error of measurement" OR sensitive OR "ceiling effect" OR "floor effect" OR irt OR rasch OR "Differential item functioning" OR dif OR "item bank" OR "cross-cultural equivalence" OR adaptation OR "Cross-Cultural Adaptation" OR translati* OR translation-back OR back-translation OR "back translation" OR "forward translation" OR forward-translation OR "backward translation" OR invariance OR "positive predictive value" OR "negative predictive value” AND depression OR depressive OR "Depressive Disorder" OR "Depressive symptom" OR "postpartum depression" OR "Treatment-resistant depression" OR "major depression" OR "Bipolar Disorder" OR "Adjustment Disorders" OR "Psychiatric Status Rating Scales" OR "Psychotic Affective Disorders" OR "Perry syndrome" OR "Major depressive disorder" OR disthymi* OR "mood disorder" OR dysphoria OR melancholia OR "mourning syndrome" OR pseudodementia OR "mourning syndrome" OR "seasonal affective disorder" OR "depressive psychosis" OR "premenstrual dysphoric disorder" OR "mood disorder" OR "Depression Anxiety" OR "puerperal depression" OR "Adjustment Disorders" OR "maternal depression" OR "late life depression" OR "Depression Scale" OR "antepartum depression" OR "Depression, Postpartum" OR "Depressive Disorder, Treatment-Resistant" OR "Depressive Disorder, Major" OR "Affective Disorders, Psychotic" OR "Major Depressive Disorder 1" OR "Major Depressive Disorder 2" AND Peruvian OR Peruvians OR Peru

**Anexo 2.**

**Figura 1.** Diagrama de flujo que esquematiza el procedimiento de selección de las publicaciones científicas encontradas en las bases de datos Scopus, Web Of Science, SciELO y PubMed


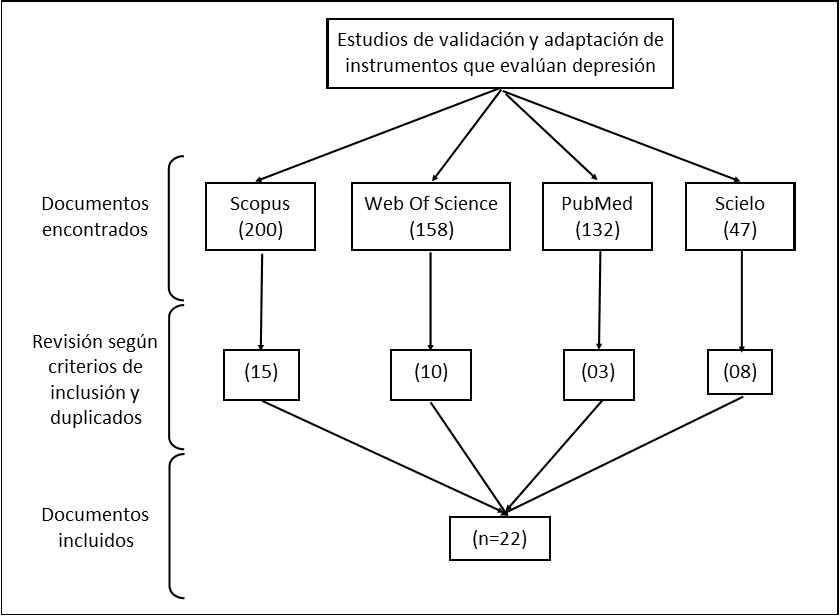

Supplement: Supplementary files. — Supplementary material. [file rpmesp-39-03-11197-s001.docx]
