# Supplementary material for: Scientific output on validation and adaptation of depression screening instruments in peruvian population
Source: Rev Peru Med Exp Salud Publica. 2022 Sep 30;39(3):357–61. doi: 10.17843/rpmesp.2022.393.11197 (PMC11397671; doi:10.17843/rpmesp.2022.393.11197)
Supplement: Translation

# Translation: Producción científica sobre validación y adaptación de instrumentos de tamizaje de la depresión en población peruana

**Authors:** Julio Cjuno, Andrea Moya, Eliana Calderón-Pérez, Cintia Quispe-Ilizarbe, Luis Mayon, José Livia

**Affiliations:** 1 Universidad Peruana Unión, Escuela de Posgrado, Lima, Peru.; 2 Universidad Peruana Unión, Escuela de Psicología, Lima, Peru.; 3 Universidad Nacional Federico Villarreal, Facultad de Psicología, Lima, Peru.

**Keywords:** Depresión, Trastorno Depresivo, Validación, Psicometría, Perú

## Abstract

El objetivo de la investigación fue describir la producción científica sobre estudios psicométricos de instrumentos de tamizaje para la depresión en población peruana. Para ello, se realizó un estudio descriptivo de la literatura científica en Scopus, Web of Science, PubMed y SciELO, con descriptores para depresión, propiedades psicométricas y Perú. Luego del proceso de revisión se incluyeron 22 estudios. Se encontraron validaciones de instrumentos de tamizaje en adultos mayores, adultos, adultos con depresión, mujeres adultas, mujeres embarazadas, profesionales de salud, estudiantes universitarios, estudiantes de secundaria y niños. El Patient Health Questionnaire (PHQ-9) fue el instrumento más estudiado. A pesar de que los estudios psicométricos cubren la mayoría de las poblaciones, quedan pendientes los pueblos originarios y poblaciones clínicas. El PHQ-9, por sus características, podría implementarse en las políticas de salud mental del Perú.

## INTRODUCCIÓN

La Organización Mundial de la Salud ^1^ señala que para el 2030 la depresión será la principal causa de morbilidad a nivel mundial. La proporción de la población mundial con este problema de salud mental se estima en 4,4%, siendo más común en mujeres que en hombres ^2^. En el Perú se ha reportado que el 15,6% de personas que se atienden en hospitales presentan algún nivel de depresión ^3^. Es reconocido como un problema de salud pública ^4^ y tiene un impacto significativo sobre la calidad de vida de las personas ^8^, por esta razón los sistemas de salud deben implementar o reforzar sus servicios profesionales para ofrecer tratamientos psicológicos e instrumentos adaptados para su tamizaje.

Las herramientas de detección y prevención son útiles para identificar con precisión a los pacientes con trastornos depresivos e iniciar un tratamiento adecuado ^5^, existen varias herramientas de detección para la depresión ^6^, pero deben ser seleccionados en base a aspectos metodológicos en términos de validez y fiabilidad, considerando los avances de la teoría de la medición y sus fundamentos teóricos ^7^. En este contexto, conocer los aspectos de la producción científica de los estudios psicométricos, respecto a los instrumentos de tamizaje de la depresión en el contexto peruano, resulta importante porque muestra el proceso de calibración de los instrumentos en los diferentes grupos etarios, regiones geográficas y contextos culturales; sobre todo cuando se trata de un país con una amplia diversidad cultural, de idiomas y lenguas nativas ^8^. Frente a ello, se propuso efectuar un estudio con el objetivo de describir la producción científica de los estudios psicométricos de los instrumentos sobre depresión adaptados o validados en el contexto peruano.

**MENSAJE CLAVE**

Motivación para realizar el estudio: es necesario conocer los instrumentos sobre depresión adaptados o validados en el Perú para planificar programas de detección, prevención y tratamiento de la depresión.

Principales hallazgos: se encontraron 22 estudios realizados en estudiantes universitarios, personal de salud, pacientes con depresión, mujeres embarazadas, adultos, adultos mayores y niños. No se hallaron estudios en idiomas originarios y en poblaciones clínicas.

Implicancias: las políticas de atención, evaluación y seguimiento de la depresión deben tomar en cuenta a los estudios psicométricos, para una práctica basada en la evidencia.

## EL ESTUDIO

La presente investigación es descriptiva. La búsqueda de literatura científica se efectuó entre 1993 hasta agosto del 2022, en PubMed, Web Of Science, Scopus y SciELO, considerando estudios en inglés o español. La estrategia de búsqueda incluyó términos y descriptores para depresión ^9^, propiedades psicométricas y población peruana; el sistema fue validado por un experto en bibliometría para una mejor precisión en la búsqueda (Material suplementario).

La búsqueda fue realizada por cuatro revisores independientes, los resultados se organizaron en Microsoft Excel 2016. Se encontraron 200 resultados en Scopus, 158 en Web Of Science, 132 en PubMed y 47 en SciELO. Se revisó el título y resumen, buscando incluir estudios que tuvieron como objetivo validar o adaptar instrumentos de evaluación de la depresión en poblaciones peruanas, ya sea como objetivo principal o secundario. Se seleccionaron artículos originales, originales breves, comunicaciones cortas y cartas al editor. Fueron elegibles 15 estudios de Scopus, 10 de Web Of Science, ocho de SciELO (colección central) y tres de PubMed. Luego de eliminar los elementos duplicados se seleccionaron 22 estudios. No se incluyeron trabajos considerados como literatura gris (tesis de pregrado, posgrado y estudios publicados en revistas con indización diferente a las bases de datos en mención) (Material suplementario).

Una vez obtenida la lista de artículos seleccionados, se realizó el análisis del documento completo y se recopilaron los datos. Las variables consideradas fueron: año de publicación, tipo de artículo, idioma, cuartil de la revista según Scimago Journal & Country Rank en la fecha de recolección de los datos, país del autor corresponsal, filiación del autor corresponsal, ciudad del autor corresponsal peruano, profesión del autor corresponsal peruano al momento de recolección de datos según el registro de grados y títulos de la Superintendencia Nacional de Educación Superior Universitaria (SUNEDU), financiamiento del estudio, producción científica por periodos de diez años, población en el que fue evaluado el instrumento, idioma de origen e idioma de destino en las traducciones de los instrumentos y propiedades psicométricas evaluadas. 

El análisis estadístico fue descriptivo, se estimaron frecuencias y porcentajes por tratarse de variables categóricas. Se utilizó el programa estadístico SPSS versión 26. 

En cuanto a los aspectos éticos, se respetó el principio de la integridad científica, debido a que analizó artículos científicos publicados en revistas arbitradas. 

## HALLAZGOS

En la producción científica sobre validación o adaptación de instrumentos que evalúan depresión en población peruana, 12 artículos (54,6%) fueron publicados en español; 20 (91,0%) fueron artículos originales; 9 (40,9%) se publicaron en revistas Q1 y 10 (45,0%) en revistas de habla inglesa; asimismo, 15 (68,2%) estudios fueron liderados por investigadores peruanos, de los cuales 5 (22,8%) tienen como filiación a la Universidad Peruana Cayetano Heredia; 13 (59,1%) investigadores corresponsales peruanos residen en Lima, donde médicos con especialidad en psiquiatría (5) y psicólogos (5) asumieron el liderazgo representando cada uno el 22,7%. Además, 15 (68,2%) estudios provienen de proyectos de investigación autofinanciados por los investigadores; la mayoría de artículos 16 (72,7%) fueron publicados entre el 2013 y 2022, y 17 (77,2%) estudios se publicaron en revistas internacionales (Tabla 1).

**Tabla 1:** Características de la producción científica psicométrica sobre depresión en Perú (n=22).

Variables	n	%
Idioma del documento		
Español	12	54,6
Inglés	9	40,9
Inglés y español	1	4,5
Tipo de documento		
Artículo original	20	91,0
Original breve	1	4,5
Carta al editor	1	4,5
Cuartil de revista ^a^		
Q1	9	40,9
Q3	3	13,6
Q2	2	9,1
Q4	2	9,1
Sin cuartil	6	27,3
País del autor corresponsal		
Perú	14	63,7
Estados Unidos	6	27,3
Colombia	1	4,5
Noruega	1	4,5
Filiación del autor corresponsal		
Universidad Peruana Cayetano Heredia	5	22,8
Harvard School of Public Health	4	18,3
Universidad de San Martín de Porres	3	13,7
Universidad de Boston	2	9,1
Universidad Privada del Norte	2	9,1
Instituto Nacional de Salud del Perú	1	4,5
Temple University	1	4,5
Universidad Católica Santo Toribio de Mogrovejo	1	4,5
Universidad Científica del Sur	1	4,5
Universidad Nacional Mayor de San Marcos	1	4,5
Universidad Norbert Wiener	1	4,5
Ciudad del autor corresponsal peruano		
Lima	13	59,1
Chiclayo	1	4,5
Cajamarca	1	4,5
Autor corresponsal con otra nacionalidad	7	31,9
Profesión del autor corresponsal peruano		
Médico con especialidad en psiquiatría	5	22,7
Psicólogo	5	22,7
Médico con otra especialidad	3	13,6
Médico maestría en epidemiología	2	9,1
Autor corresponsal con otra nacionalidad	7	31,9
Financiamiento nacional o internacional		
No	15	68,2
Sí	7	31,8
Producción científica por periodos de 10 años		
2013 al 2022	16	72,7
2003 al 2012	5	22,8
1993 al 2002	1	4,5

a Según el Scimago Journal & Country Rank 2022

Las validaciones o adaptaciones se realizaron en adultos (4 de 22); adultos con depresión (3 de 22); mujeres adultas (4 de 22); mujeres embarazadas (4 de 22); profesionales de salud (1 de 22); estudiantes universitarios (2 de 22); estudiantes de secundaria (1 de 22), y niños (1 de 22); además, 17 (72,2%) de las validaciones o adaptaciones fueron realizadas en muestras no clínicas, y la mayor proporción de los instrumentos (13 de 22) se tradujeron del inglés al español (Tabla 2).

**Tabla 2:** Población donde se realizó la adaptación e idioma de origen y destino de traducción.

Variables	n	%
Población en el que fue evaluado el instrumento		
Adultos	4	18,3
Mujeres adultas	4	18,3
Mujeres embarazadas	4	18,3
Adultos con depresión	3	13,6
Estudiantes universitarios	2	9,0
Adultos mayores	1	4,5
Estudiantes de secundaria	1	4,5
Niños	1	4,5
Profesionales de salud	1	4,5
Jóvenes, adultos, adultos mayores	1	4,5
Idioma origen e idioma destino (traducción)		
Inglés - español (Perú)	13	59,1
Construido en español (Perú)	6	27,3
Español (España) - español (Perú)	2	9,1
Español (Colombia) - español (Perú)	1	4,5

Los estudios psicométricos analizaron, mayormente la fiabilidad (20 de 22); validez de constructo (20 de 22); sensibilidad y especificidad (15 de 22) (Tabla 3). Por otro lado, la mayor proporción de los trabajos (9 de 22) adaptó la *Patient Health Questionnaire* (PHQ-9) en estudiantes universitarios, personal de salud, mujeres embarazadas, adultos y adultos mayores (Tabla 4).

**Tabla 3:** Propiedades psicométricas evaluadas en los instrumentos que miden la depresión en Perú.

Propiedades psicométricas	Sí		No	
	n	%	n	%
Validez de constructo	20	90,9	2	9,1
Fiabilidad	20	90,9	2	9,1
Sensibilidad y especificidad	15	68,2	7	31,8
Validez de contenido	9	40,9	13	59,1
Análisis de invarianza	4	18,3	18	81,7

**Tabla 4:** Instrumentos adaptados para evaluar depresión en Perú.

Instrumentos ^a^	n
Patient Health Questionnaire (PHQ-9)	9
Edinburgh Postnatal Depression Scale (EPDS)	2
Inventario de depresión de Beck (BDI-II)	2
State-Trait Depression Inventory (ST-DEP)	2
Patient Health Questionnaire (PHQ-2)	1
Escala de depresión Geriátrica GDS-4	1
General Health Questionnaire (GHQ-12)	1
Children’s Depression Inventory-short (CDI)	1
The Self-Reporting Questionnaire (SRQ)	1
Depressive Psychopathology Scale (DPS)	1
Center for Epidemiological Studies Depression Scale (CES-D)	1
Zung Self-Rating Depression Scale (ZSDS)	1

a Existen 23 instrumentos de tamizaje en el contexto peruano porque algunos estudios validaron dos instrumentos y los compararon.

## DISCUSIÓN

En los últimos diez años (2013 - 2022) se encontraron la mayor proporción estudios de adaptación o validación de instrumentos de tamizaje para la depresión, en revistas del cuartil 1, de habla inglesa. La preferencia por revistas internacionales, de habla inglesa y del cuartil 1 puede deberse a que los investigadores buscan la mayor visibilidad e impacto de sus trabajos en la comunidad científica ^10^. 

La elección de revistas científicas internacionales posiblemente sea porque las publicaciones científicas peruanas aún no logran ubicarse dentro del primero o segundo cuartil ^11^. Sin embargo, se ha incrementado el interés en la adaptación y validación de las pruebas de tamizaje de la depresión; posiblemente impulsado por las políticas de calidad de las universidades peruanas que iniciaron la contratación de investigadores como parte del *staff* de docentes ^12^ y el proceso de licenciamiento.

Se han realizado validaciones en poblaciones de adultos mayores, adultos, adultos con depresión, mujeres adultas, mujeres embarazadas, profesionales de salud, estudiantes universitarios, estudiantes de secundaria y niños; reflejando la diversidad de los grupos etarios atendidos. Una tarea pendiente es adaptar o construir instrumentos en idiomas nativos u originarios, debido a que nuestro país, por su riqueza cultural y lingüística, posee más de 50 lenguas originarias ^13^, una diferencia cultural y lingüística que parte de la misma construcción teórica de cómo esa población percibe la depresión, muestra la necesidad de instrumentos adaptados al contexto ^7^. Otra razón para promover la adaptación de instrumentos en los pueblos originarios es la prevalencia de depresión en pobladores quechuas, que alcanza un 38,9% ^9^, que es siete veces mayor que la prevalencia mundial en adultos ^1^, por lo que sería una necesidad y tarea pendiente. 

Una mayor proporción de los estudios mostraron los análisis de fiabilidad, validez de constructo, sensibilidad/especificidad y validez de contenido. La validez y fiabilidad de los estudios evidencia que se están siguiendo los procesos estadísticos y psicométricos adecuados para la adaptación y construcción de instrumentos ^7^ ya que la validez de contenido permite adaptar o corregir la redacción al contexto cultural-lingüístico y evalúa la pertinencia del contenido relacionado al constructo teórico ^14^. La validez de constructo analiza las factores que explican el constructo a partir de su estructura interna y la fiabilidad determina qué tan preciso son las puntuaciones del instrumento bajo determinadas condiciones, mientras que el análisis de especificidad y sensibilidad determina la capacidad diagnóstica con el que un instrumento calificará eficientemente entre participantes que tienen o no el problema que se evalúa ^14^. 

Por la diversidad biosociodemográfica de la población peruana es necesario contar con instrumentos que no impliquen sesgos, por ello, el análisis de invarianza es importante; aunque por ahora es la característica de pocos instrumentos. Este procedimiento verifica si el instrumento pudiera o no ser transversal a las características de la población en estudio como grupos etarios, sexo, lugar de residencia, poblaciones clínicas/no clínicas y otras similares ^7^. Por lo que, no todos los instrumentos estarían habilitados para su uso en diferentes poblaciones o grupos.

 La mayor proporción de estudios de adaptación o validación analizaron el PHQ-9 y es posible su uso en estudiantes universitarios, personal de salud, adultos de ambos sexos, mujeres embarazadas en la atención primaria de la salud y en adultos mayores. Esta preferencia puede deberse a sus adecuadas propiedades psicométricas y adaptaciones en más de 20 idiomas, siendo el instrumento de tamizaje más utilizado a nivel mundial y elegido en un consenso internacional entre 15 escalas evaluadas para el tamizaje de la depresión ^15^. En Perú, cuenta con una adaptación cultural por jueces expertos y posteriormente se ha realizado el análisis de la validez, fiabilidad e invarianza ^16^, que determina la elegibilidad del PHQ-9 sobre otros instrumentos.

En ese contexto, los organismos peruanos responsables del cuidado de la salud mental podrían implementar el PHQ-9 en sus guías de atención de la depresión en las poblaciones donde ya cuenta con sólidas evidencias psicométricas. Sobre todo, porque algunos documentos oficiales como la guía técnica de cuidados de salud mental del personal de salud en el contexto de la COVID-19 del Ministerio de Salud, recomienda el cuestionario de autorreporte o *Self-Reporting Questionnaire* (SRQ) ^17^ que, a la fecha, fue validado solo en mujeres, mientras que para el tamizaje en la atención primaria de la salud se recomienda a la escala de *Hamilton Depression Rating Scale* (HDRS) ^18^, sin evidencias de validación en Perú y en la Norma Técnica de Salud para la atención Integral de Salud de las personas adultas mayores se implementa la Escala Abreviada de Yesavage ^19^. Este último, no recomendado para su uso por contar con validez inicial de su estructura interna, pero carece de fiabilidad ^20^. Lo anterior es preocupante, pues en la selección de instrumentos de tamizaje se debe realizar considerando las mejores evidencias de sus propiedades psicométricas. 

Como una limitación del presente estudio se debe mencionar que no se incluyeron publicaciones de revistas indizadas en otras bases de datos regionales o de la literatura gris. Sin embargo, al buscar en Scopus, Web of Science, PubMed y SciELO, se ha incorporado la mayor literatura arbitrada sobre la validez o construcción de pruebas que evalúan la depresión en la población peruana. 

En conclusión, se encontraron 22 estudios entre adaptaciones y validaciones de instrumentos que evalúan la depresión en población peruana. Se han adaptado a estudiantes universitarios, personal de salud, pacientes con depresión, mujeres embarazadas, adultos de ambos sexos, adultos mayores y niños. Queda pendiente realizar estudios instrumentales en idiomas originarios y en poblaciones clínicas. El PHQ-9 por sus características podría ser implementado en las políticas de atención, evaluación y seguimiento de la depresión en las poblaciones donde se han realizado estudios psicométricos.
